# Valorization of untreated rice bran towards bioflocculant using a lignocellulose-degrading strain and its use in microalgal biomass harvest

**DOI:** 10.1186/s13068-017-0780-6

**Published:** 2017-04-13

**Authors:** Cong Liu, Yan Hao, Jihong Jiang, Weijie Liu

**Affiliations:** School of Life Science, The Key Laboratory of Biotechnology for Medicinal Plant of Jiangsu Province, Jiangsu Normal University, No. 101, Shanghai Road, Tongshan District, Xuzhou, 221116 Jiangsu China

**Keywords:** Bioflocculant, Rice bran, Microalgae harvest, *Chlorella minutissima* UTEX2341, *Bacillus agaradhaerens*, Xylanase

## Abstract

**Background:**

Microalgae are currently considered as a promising feedstock for the production of biofuels and high-value products. However, the efficient harvest of microalgal biomasses from their culture broth is a major challenge. The harvesting of algal biomass by flocculation combined with gravity sedimentation is more convenient and cost-effective than traditional methods such as centrifugation and filtration. Compared to inorganic and chemically synthetic flocculants, bioflocculants are a suitable choice for microalgal harvest due to their biodegradable and nontoxic properties. Nonetheless, the high production costs associated with expensive substrates hinder the commercial applications of bioflocculants. Previous studies have shown that the hydrolysates of lignocellulosic biomasses from dilute acid hydrolysis can be utilized as an inexpensive carbon source for the production of bioflocculants. However, the toxic by-products generated in the dilute acid hydrolysis step limit the efficiency of subsequent fermentation. The strains that produce bioflocculants by using untreated lignocellulosic materials can circumvent the pretreatment process, as well as promote the application of bioflocculants in microalgal harvest.

**Results:**

Under alkaline fermentation conditions, the alkaliphilic strain *Bacillus agaradhaerens* C9 secreted 1.69 IU/mL of alkali-tolerant xylanase and 0.06 IU/mL of cellulase, indicating that this particular strain can efficiently convert untreated rice bran into bioflocculant (RBBF-C9), thereby circumventing rice bran pretreatment for downstream fermentation. The optimal fermentation conditions that result in the highest bioflocculant yield (12.94 g/L) were as follows: 20 g/L of untreated rice bran, 3 g/L of yeast extract, and 20 g/L of Na_2_CO_3_ at 37 °C for 24 h. RBBF-C9 contained 74.12% polysaccharides and 4.51% proteins, and was estimated to be 137 kDa. Furthermore, the bioflocculant RBBF-C9 exhibited good flocculating efficiency (91.05%) of oil alga *Chlorella minutissima* UTEX2341 when 60 mg/L of RBBF-C9 was added into the algal culture broth.

**Conclusions:**

This study demonstrated that untreated rice bran is a suitable inexpensive substrate for the production of bioflocculants, and thus provides a novel approach in utilizing rice bran. The extracted bioflocculants may be potentially used in biomass harvesting of the oil algae *C. minutissima* UTEX2341 from the culture broth.

**Electronic supplementary material:**

The online version of this article (doi:10.1186/s13068-017-0780-6) contains supplementary material, which is available to authorized users.

## Background

Microalgae have recently been considered as an attractive and renewable feedstock not only for biofuel production [1–3], but also for the production of many value-added products such as nutritional supplements, pigments, steroids, cosmetics, and pharmaceuticals [4–7] because they exhibit a higher growth rate and a shorter growth period, and requires less water and land area compared to traditional crops [8, 9]. However, harvesting and dewatering of microalgal biomass from the culture broth remains a major challenge due to their small cell size (3–20 μm), low biomass concentration, and colloidal stability of microalgal cells [9, 10]. It has been estimated that the cost of biomass harvesting generally accounts for more than 30% of the total production cost of microalgal biofuels. Various harvesting strategies have been developed to separate the microalgae cells from their culture broth, including centrifugation, air flotation, ultrasound, filtration, flocculation, electrolytic method, magnetic coagulant, gravity sedimentation [1, 5, 11–14], and bio-flocculation based on algal/bacterial, algal/fungal, or algal/algal interactions [15, 16]. Centrifugation, air flotation, and ultrasound are not economical for large-scale harvest of microalgae due to the requirement of high energy inputs [17]. Filtration is only suitable for the large multicellular microalgae, and the frequent filter changes increase its operational complexity [18]. Magnetic coagulant and electrolytic method are reported to be able to enhance the harvest efficiency of certain microalgal species; however, they are also expensive and will contaminate the microalgae biomass [19]. Although fungal-assisted algae harvest is found to be effective for some microalgae, it is a challenge for this method to separate the fungi and microalgal cells. And the harvest method based on mixing self-flocculating and non-flocculating microalgae is only suitable for specific microalgae [15]. Flocculation is known as one of the inexpensive strategies for microalgae harvesting [20], which can increase the aggregation size of microalgae, and thus enhance the efficiency of gravity sedimentation or flotation [19].

Flocculating agents are generally classified into three main groups: inorganic flocculants such as poly-aluminum chloride and aluminum sulfate, organic polymeric flocculants such as polyethyleneimine, and bioflocculants that are a complex mixture of biopolymers secreted by microorganisms during their growth and cell lysis, including polysaccharides, proteins, and lipids [21–24]. The use of organic/inorganic flocculants caused serious health and environmental problems [25, 26]. Thus, bioflocculants have received increasing attention as nontoxic and biodegradable substitutes for conventional inorganic and chemically synthetic flocculants [24, 27] in various industrial fields such as wastewater treatment and microalgae harvest [28–34]. However, the commercial applications of bioflocculants are often limited by their high production cost due to the use of expensive substrates [35, 36]. To reduce production costs, activated sludges or various wastewaters such as potato starch wastewater and phenol-containing wastewater are used as inexpensive culture media for the production of bioflocculants [37–41]. Various lignocellulosic biomasses such as peanut hull, rice hull, and corn stover whose hydrolyzates from dilute acid hydrolysis have been used as inexpensive carbon sources for the production of bioflocculants [42–44]. However, toxic by-products, including furan derivatives, organic acids, phenolic compounds, and lignin derivatives, are generated during the dilute-acid hydrolysis process [45, 46]. These toxins not only severely inhibit fermentation efficiency, but also influence the safety of the bioflocculant products. The pH neutralization and detoxification steps are often part of the process of generating dilute acid hydrolysates prior to downstream fermentation [47]. Therefore, bioflocculant production using a strain that can secrete lignocellulolytic enzymes and thereby produce bioflocculants by directly degrading untreated lignocellulosic materials is a promising strategy because it integrates enzyme production, enzymatic saccharification of lignocellulosic biomass, and fermentation into a single process [48–50]. In a previous study, *Cellulosimicrobium cellulans* L804 has been reported as a lignocellulose-degrading strain that could convert untreated corn stover into bioflocculant [51]. However, the optimal pH for activities of lignocellulolytic enzymes secreted by *C. cellulans* L804 is around 5.2–6.0, which largely varies from its optimal fermentation pH (pH 8.2) for bioflocculant production, thereby decreasing the conversion efficiency of corn stover into bioflocculant due to low enzyme activity at an unsuitable pH.

Rice bran is a by-product of the rice milling industry with world production of 50–60 million each year [52]. However, its industrial applications are limited to its use as animal feed, pollutant adsorbent, or for the production of rice bran oil [53]. The amount of rice bran available is far in excess of its local applications, thereby frequently leading to disposal problems [54]. Rice bran mainly contains carbohydrates, proteins, fats, and minerals, and the presence of a high amount of carbohydrates (cellulose and hemicellulose) makes it an alternative inexpensive feedstock for the conversion into value-added microbial products [55].

The present study has shown that under alkaline conditions, *B. agaradhaerens* C9 secretes alkali-tolerant lignocellulolytic enzymes and thereby directly converts untreated rice bran into bioflocculant (named as RBBF-C9). High conversion efficiency was achieved due to the similarity between the optimal pH range for bioflocculant fermentation and enzyme activities. The highest bioflocculant yield (12.94 g/L) was obtained within 24 h. Moreover, the obtained bioflocculant RBBF-C9 was applied in harvesting the oil algae *C. minutissima* UTEX2341.

## Results and discussion

### Selection of lignocellulose biomass for bioflocculant production

**Fig. 1:**
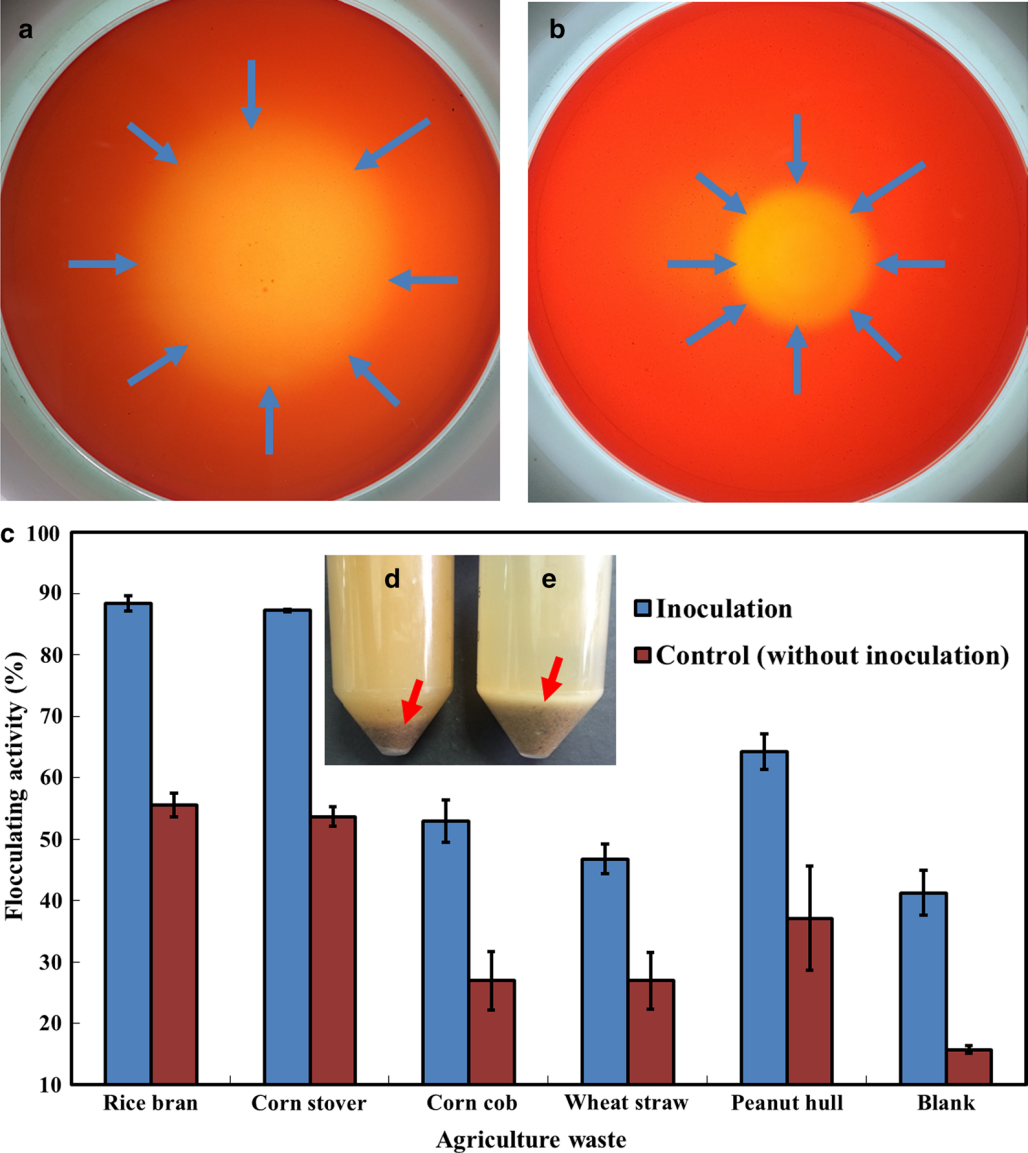
Xylanase (**a**) and cellulase (**b**) of *B. agaradhaerens* C9 were evaluated using agar plates containing xylan or CMC. Images were captured at 48 h of culture. **c** Production of RBBF-C9 when different agricultural wastes were used as carbon source. *B. agaradhaerens* C9 was grown in the media containing 3 g/L yeast extract and different biomasses, and in the control media without inoculation of *B. agaradhaerens* C9. The medium added with 3 g/L yeast extract but without biomasses was used as blank. 100-μL fermentation broth was collected at 24 h of culture and used in the flocculating activity assay. The residues of lignocellulosic biomasses in the broths with (**d**) and without (**e**) inoculation of *B. agaradhaerens* C9. Images were captured at 24 h of culture

Agricultural wastes, whose hydrolyzates generally contain glucose and xylose, are abundantly available in China. Therefore, the strain that can secrete lignocellulolytic enzymes and thereby produce bioflocculants by directly degrading untreated agricultural wastes may be an effective approach in reducing the production cost of bioflocculants. A previous study described *B. agaradhaerens* C9 as a highly active polysaccharide bioflocculant-producing strain under alkaline conditions [56]. To test whether *B. agaradhaerens* C9 can utilize untreated lignocellulosic biomasses as carbon source, the lignocellulolytic enzymes of *B. agaradhaerens* C9 were analyzed in this study. Figure 1a, b shows the clearing zones surrounding the colonies that were grown on the agar plates that contained xylan and cellulose, indicating that *B. agaradhaerens* C9 secretes xylanase and cellulase. Therefore, *B. agaradhaerens* C9 may be potentially used in the production of bioflocculants by directly degrading and utilizing untreated lignocellulosic biomasses.

Five kinds of untreated lignocellulosic biomasses, including rice bran, corn stover, corn cob, wheat straw, and peanut hull were directly used as the sole carbon source of fermentation media. Figure 1c shows that >87% flocculating activity was achieved when rice bran and corn stover were used as carbon sources, which is significantly higher than that using corn cob, wheat straw, and peanut hull. On the other hand, the control broth (without inoculation of *B. agaradhaerens* C9) showed relatively low flocculating activity, which might be attributable to the release of macromolecular substances such as pectin from the lignocellulosic biomasses under alkaline conditions. The flocculating activities of all fermentation broths with five kinds of lignocellulosic biomasses were significantly higher than that of the control broth without inoculation, and fewer residues of lignocellulosic biomasses were observed after fermentation by *B. agaradhaerens* C9 compared to that of the control broths (Fig. 1d, e), thereby suggesting that the bioflocculant products were mainly produced from the conversion of lignocellulosic biomasses by *B. agaradhaerens* C9. The flocculating activity of the blank broth (without adding lignocellulosic biomasses) was much lower than that of the broths with lignocellulosic biomasses, and no bioflocculant product was obtained from the blank broth, further indicating that the bioflocculant products were mainly from the conversion of lignocellulosic biomasses by *B. agaradhaerens* C9. Moreover, the highest flocculating activity of 88.42% and a yield of 3.67 g/L were observed when rice bran was used as carbon source, which is slightly higher than the flocculating activity of 87.24% and the yield of 3.65 g/L that was achieved using corn stover as carbon source. Based on these findings, untreated rice bran was selected for the subsequent studies.

### Effects of various rice bran dosages on the flocculating activity and yield of RBBF-C9

**Fig. 2:**
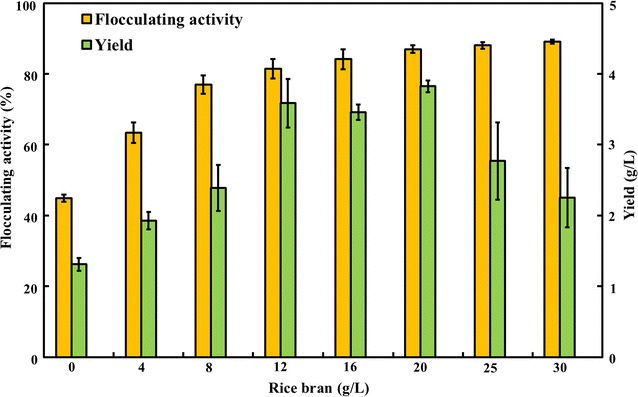
Effects of rice bran concentrations (3 g/L of yeast extract was used as nitrogen source) on the flocculating activity and yield of RBBF-C9. The 24-h fermentation broth was sampled for analysis

The ratio of the carbon source to nitrogen source significantly influences cell growth and bioflocculant production. The optimal carbon/nitrogen ratio leads to the highest flocculating activity within the shortest culture time [24]. Figure 2 shows the effects of various rice bran dosages on RBBF-C9 production when 3 g/L of yeast extract was added as nitrogen source. An enhancement of flocculating activity and yield was observed with increasing rice bran dosages from 0 to 20 g/L, and a flocculating activity of 87% and the highest bioflocculant yield of 3.83 g/L were achieved when *B. agaradhaerens* C9 was cultured in the medium containing 20 g/L of rice bran. A further increase in the rice bran dosage inhibited RBBF-C9 production, which may be due to limitations in oxygen transfer in the fermentation medium with excessive amounts of solid rice bran, thereby influencing cell growth and enzymatic secretion of *B. agaradhaerens* C9.

### Effects of Na_2_CO_3_ concentrations on the flocculating activity and yield of RBBF-C9

**Fig. 3:**
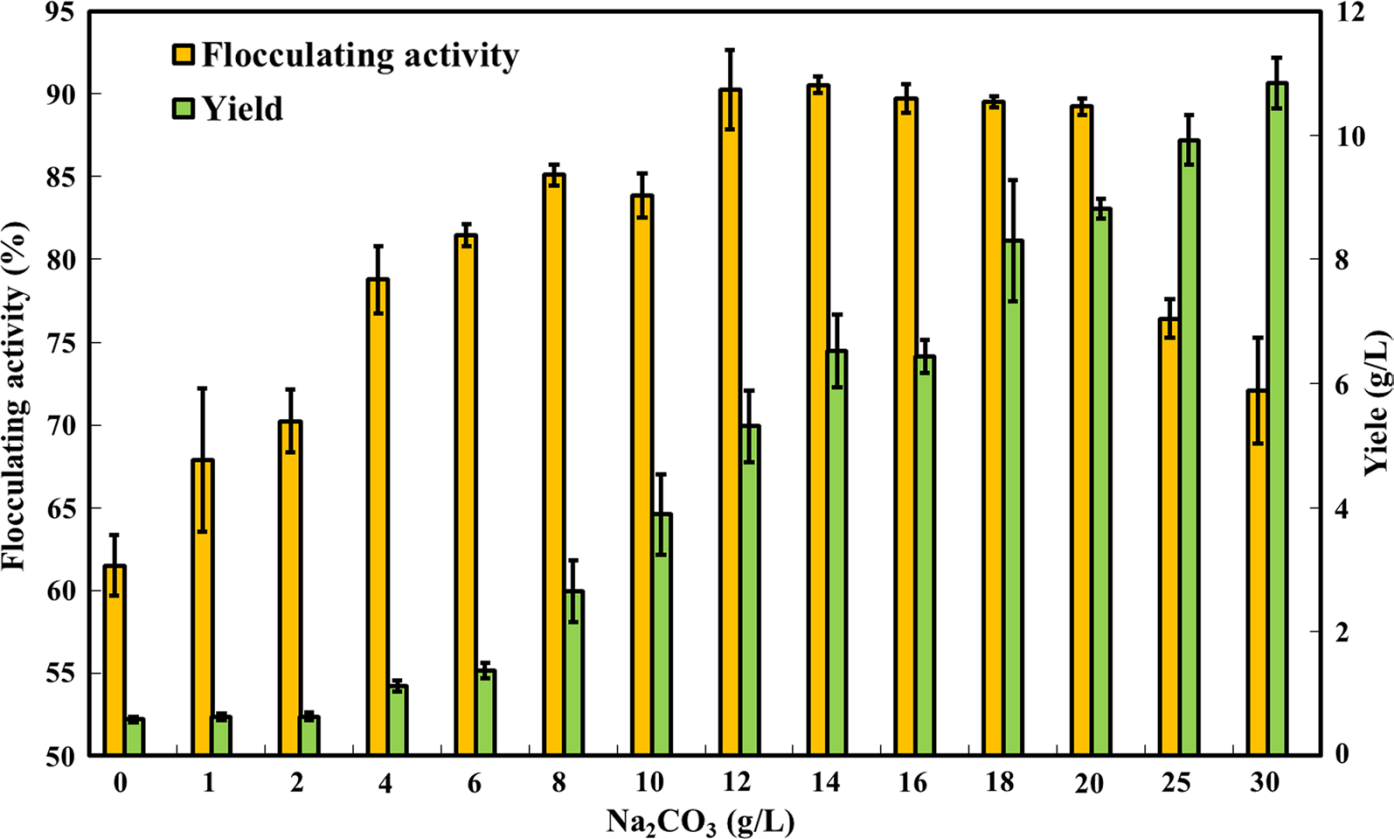
Effects of Na_2_CO_3_ concentrations on the flocculating activity and yield of RBBF-C9. The 24-h fermentation broth was collected for analysis. The medium contained 20 g/L of untreated rice bran and 3 g/L of yeast extract. *Error bars* indicate the standard deviation of three replicates

The initial pH of the fermentation medium influences the charge of the molecules on the cell surface, oxidation–reduction potential, microbial nutriment assimilation, and enzyme reactions [24, 57]. A previous study showed that under the alkaline conditions using Na_2_CO_3_ to adjust pH, *B. agaradhaerens* C9 produces the bioflocculant because it could buffer changes in pH levels [56]. Thus, the effects of various Na_2_CO_3_ concentrations on the flocculating activity and yield of RBBF-C9 were analyzed. Figure 3 shows that the flocculating activity and yield of RBBF-C9 improved with increasing Na_2_CO_3_ concentrations from 0 to 20 g/L. However, a further increase in the Na_2_CO_3_ concentration eventually resulted in a decrease in flocculating activity. Thus, the optimal Na_2_CO_3_ concentration of 20 g/L was selected to promote the enzymatic hydrolysis of rice bran, and simultaneously provide a buffer for the production of the acidic polysaccharide bioflocculant RBBF-C9. Previous studies have shown that alkaline pretreatment could cause spelling of lignocellulosic substances, thereby resulting in an increase in internal surface area, and the separation of structural linkages between lignin and carbohydrates [47]. Thus, alkaline fermentation conditions could increase the conversion efficiency of lignocellulosic biomasses into valuable products [51].

### The optimal pH range for the activities of xylanase and cellulase

**Fig. 4:**
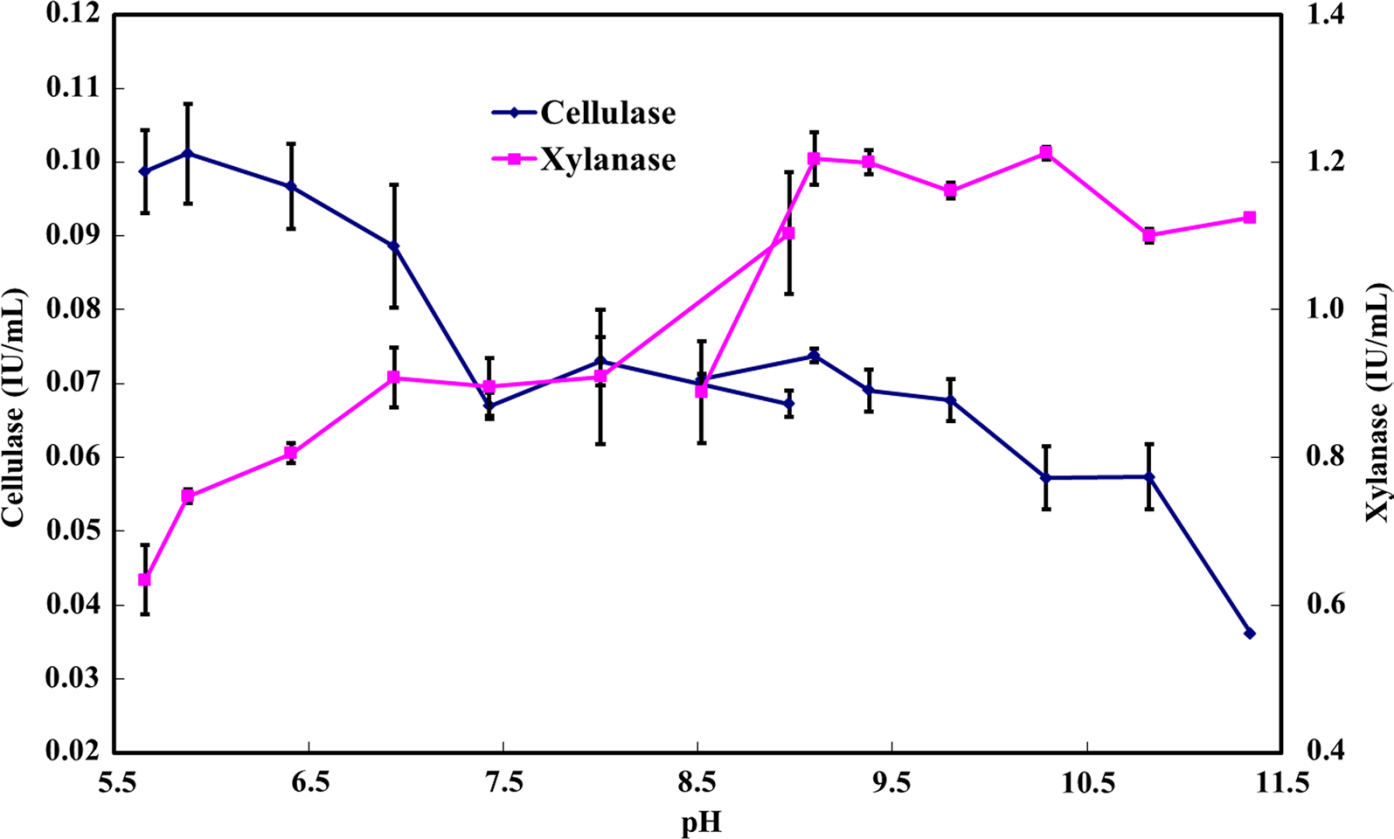
The activities of xylanase and cellulase secreted from *B. agaradhaerens* C9 were determined in 0.2 M phosphate buffer (pH 5.6–8.9) and 0.2 M NaHCO_3_–Na_2_CO_3_ buffer (pH 8.5–11.3) at 50 °C. *Error bars* represent the standard deviation of three replicates

**Fig. 5:**
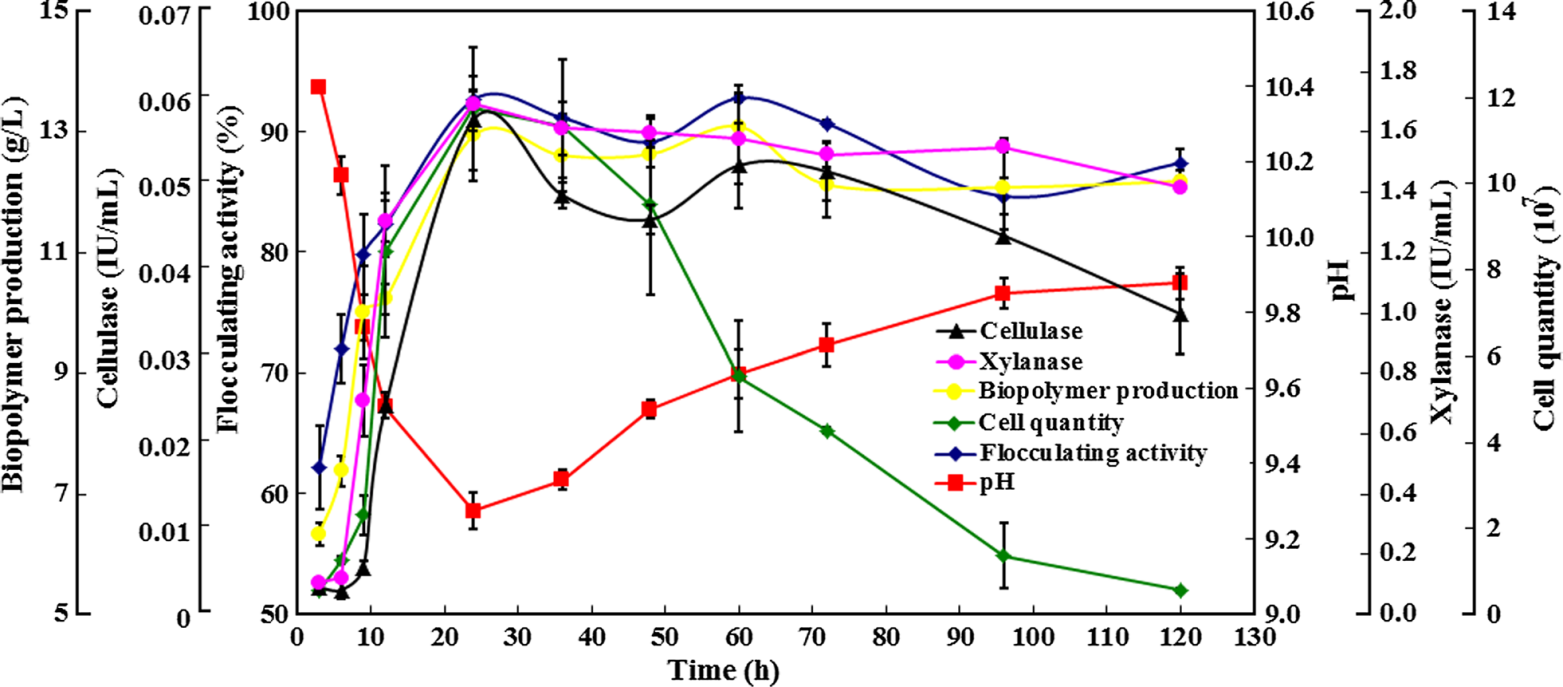
Time curves of xylanase, cellulase, pH, and RBBF-C9 production during cell growth in the optimized fermentation medium with 180 rpm shaking at 37 °C. *Error bars* represent the standard deviation of three replicates

A previous study has shown that under weak alkaline conditions (pH 8.2), *C. cellulans* L804 produces bioflocculants by using untreated corn stover. However, the optimal pH for the activities of both xylanase and cellulase from *C. cellulans* L804 is around 5.2–6.0 [51], which is significantly different from its optimal fermentation pH (pH 8.2) for bioflocculant production, thereby decreasing the conversion efficiency of corn stover into bioflocculant due to the low activity of these two hydrolytic enzymes at an unsuitable pH. In the present study, the effects of pH on the activities of xylanase and cellulase were analyzed. Figure 4 shows that the optimal pH range for xylanase activity was highly alkaline pH 9.1–10.3, which was similar to another alkaliphilic xylanase (optimal pH 11) that is produced by an alkaliphilic strain *Bacillus halodurans* PPKS-2 [58]. The optimal pH for activity of cellulase from *B. agaradhaerens* C9 was 5.9. Although alkaline conditions were not optimal for cellulase activity, 60–70% of the cellulase activity relative to its highest activity remained within the alkaline pH range of 9.1–10.8. The optimal fermentation pH range for *B. agaradhaerens* C9 was 9.3–10.4 (Fig. 5), which was similar to the optimal pH range for the activity of xylanase that is secreted by *B. agaradhaerens* C9. Thus, compared to the reported strain *C. cellulans* L804, *B. agaradhaerens* C9 shows higher efficiency in converting the lignocellulosic biomasses into bioflocculants.

### Time profiles for cell growth, xylanase, cellulase, pH, and RBBF-C9 production

The time curves for cell growth, cellulase, xylanase, pH, and RBBF-C9 production were analyzed when *B. agaradhaerens* C9 was cultured in a medium that was supplemented with rice bran as carbon source. Figure 5 shows that the flocculating activity and yield of bioflocculant RBBF-C9 sharply increased from 6 to 24 h, which is almost parallel to its cell growth, thus indicating that RBBF-C9 is mainly produced by *B. agaradhaerens* C9 and does not result from the release of macromolecular substances from rice bran under alkaline conditions. The highest flocculating activity (92.67%) of kaolin clay solution was achieved after 24 h of culture. After 36 h of culture, the cells entered the death phase. The drop in flocculating activity and yield of RBBF-C9 during this period may be due to deflocculation enzyme activity that is caused by cell lysis [36, 59]. The pH significantly decreased from 10.4 to 9.3 in the first 24 h, which might have been caused by the production of acidic polysaccharides during cell growth [51, 56, 60]. After 24 h of culture, the increase in pH might have been due to the release of intracellular substances during cell lysis. A similar change in pH was reported in a previous study [60]. Figure 5 also shows sharp increases in xylanase and cellulase activities in the first 24 h of incubation. A positive correlation between the enzymatic activities and RBBF-C9 production was observed, thereby suggesting that the production of RBBF-C9 from rice bran was dependent on the activities of xylanase and cellulose secreted by *B. agaradhaerens* C9. The highest levels of xylanase (1.69 IU/mL) and cellulase (0.06 IU/mL) were observed within the fermentation pH range of 9.3–10.4 at 24 h of culture, which is significantly higher than the activity of xylanase (0.6 IU/mL) and cellulase (0.046 IU/mL) produced by *C. cellulans* L804 after 36 h of culture in a medium with untreated corn stover as carbon source [51], thereby indicating that *B. agaradhaerens* C9 more efficiently converts untreated lignocellulosic biomasses into bioflocculants than *C. cellulans* L804. The highest bioflocculant yield (12.94 g/L) was achieved at 24 h, which is significantly higher than that of *C. cellulans* L804 (4.75 g/L) after 48 h of culture [51].

### Properties of the bioflocculant RBBF-C9

The composition analysis indicated the presence of 74.12% polysaccharides and 4.51% proteins in RBBF-C9, suggesting that polysaccharides are the main functional components. According to the result of gel permeation chromatography (GPC), the molecular weight of RBBF-C9 was estimated to be 137 kDa. Elemental analysis showed that the mass proportion of C, H, N, and S was 35.06, 5.72, 7.21, and 3.94%, respectively. FTIR spectrum of RBBF-C9 (Additional file 1: Fig. S1) exhibited a strong broad peak around 3323 cm^−1^, which is identified as hydroxyl groups, and a weak C–H stretching peak at 2929 cm^−1^. The spectrum also displayed bands at 1655 and 1440 cm^−1^, which are contributed to carboxyl groups. The absorption peak ranged from 1000 to 1200 cm^−1^ suggests the presence of sugar derivatives. This result is consistent with the FTIR spectrum of bioflocculant produced by *B. agaradhaerens* C9 using glucose as carbon source [56], and confirms that polysaccharides are the main components of RBBF-C9.

### Application of RBBF-C9 in harvesting *C. minutissima* UTEX2341 cells

*Chlorella minutissima* is currently considered as a promising feedstock for biofuel production due to its capability for rapid growth and its high lipid content. Significant environmental factors that influence the growth of *C. minutissima* UTEX2341 have been previously investigated [61, 62]. In addition, the culture conditions of *C. minutissima* UTEX2341 were optimized to enhance its cell growth and lipid production [63, 64]. Flocculation is a suitable strategy for harvesting *C. minutissima* UTEX2341 biomass. Inorganic flocculants such as aluminum, ferric, and zinc salts have been used to flocculate the *C. minutissima* biomass, and the optimum concentration was 0.75 g/L for their sulfate salts. However, inorganic flocculants are harmful to microalgal cells. For example, aluminum salts induce cell lysis, and ferric salts resulted in changes in the color of microalgal cells [65].

**Fig. 6:**
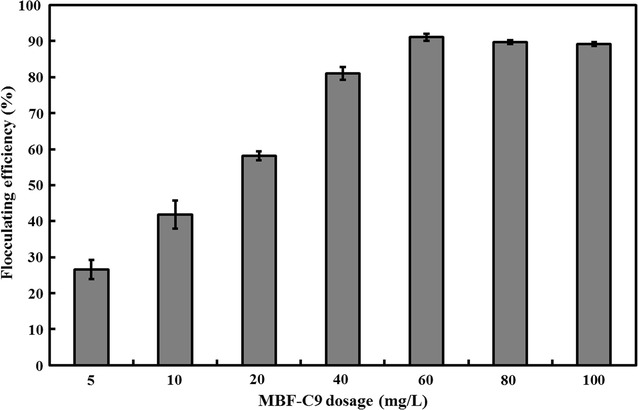
Flocculating efficiency of *C. minutissima* UTEX2341 at different RBBF-C9 dosages

In the present study, the feasibility of harvesting *C. minutissima* UTEX2341 cells using bioflocculant RBBF-C9 was evaluated. Figure 6 shows the flocculating rates of *C. minutissima* UTEX2341 when different concentrations of RBBF-C9 were added into the algal broth. A positive correlation between the flocculating activity and RBBF-C9 dosage was observed when RBBF-C9 dosage varied from 5 to 60 mg/L. The highest flocculating efficiency of 91.05% was achieved at the optimal dosage of 60 mg/L. A further increase in RBBF-C9 dosage caused a slight decrease in the harvest efficiency of microalgal biomass. Similar trends were observed in previous studies [51, 56]. This slight decrease could be explained by the excessive addition of negatively charged polysaccharide bioflocculants that induced the repulsion between similarly charged particles.

The flocculating efficiency of microalgae by bioflocculant is dependent on their species. The cell sizes and cell surface properties of microalgae can significantly influence their harvest efficiency. For example, the flocculating rate of bioflocculant produced by strain *C. cellulans* L804 to microalga *Chlamydomonas reinhardtii* was 99%, while only 93% to *C. minutissima* [51]. A potent bioflocculant γ-PGA produced by *Bacillus licheniformis* CGMCC2876 exhibited a highest flocculating rate of 99% to *Desmodesmus* sp. F51 [66]. The cell size of *Chlorella* sp. is smaller compared to most microalgae (only 3–8 μm). The flocculating rates of γ-PGA produced by *Bacillus subtilis* to *C. vulgaris* and *C. protothecoides* were 91 and 98%, respectively [67]. In addition, most bioflocculants used in the harvest of microalgal biomass are generally produced from expensive substrates [56]. The bioflocculant γ-PGA is produced from trisodium citrate, glycerol, and sodium glutamate [66]. In the present study, the bioflocculant RBBF-C9 was produced through directly degrading untreated agricultural by-product (rice bran), which could reduce the production cost of bioflocculant, achieve the resourceful utilization of abundantly available agricultural wastes and promote the efficient harvesting of microalga *C. minutissima* UTEX2341 cells.

## Conclusions

This study has shown that under alkaline conditions, *B. agaradhaerens* C9 secretes alkali-tolerant xylanase and cellulase, and simultaneously produces bioflocculant RBBF-C9 by directly degrading untreated rice bran. The highest yield of RBBF-C9 (12.94 g/L) was achieved at the following optimized conditions: 20 g/L of untreated rice bran as carbon source, 3 g/L of yeast extract as nitrogen source, and supplemented with 20 g/L of Na_2_CO_3_. Furthermore, RBBF-C9 exhibited the highest flocculating efficiency (91.05%) for microalga *C. minutissima* UTEX2341 cells, which thereby promotes the application of bioflocculant in the low-cost harvesting of microalgae for biofuel production.

## Methods

### Strain and culture conditions

The bioflocculant-producing strain *B. agaradhaerens* C9 was isolated [56] and deposited to China General Microbiological Culture Collection Center (CGMCC, Beijing, China) under the accession number CGMCC13057. The strain was inoculated into 25 mL of seed medium and cultured on a reciprocal shaker at 180 rpm and 37 °C. After 12 h of incubation, the obtained seed culture was used for further experiments. The composition of the seed medium was as follows: 10 g/L glucose, 10 g/L yeast extract, 1.3 g/L K_2_HPO_4_, and 0.2 g/L MgSO_4_·7H_2_O. After sterilization at 115 °C for 30 min, the medium was supplemented with 10 g/L Na_2_CO_3_. To evaluate the activity of cellulase and xylanase that were secreted by *B. agaradhaerens* C9, the seed culture was dropped onto an agar plate containing 10 g/L of CMC-Na (for cellulase assay) or oat spelt xylan (sigma) (for xylanase assay), 10 g/L of yeast extract, 1.3 g/L K_2_HPO_4_, 0.2 g/L MgSO_4_·7H_2_O, 15 g/L agar, and supplemented with 10 g/L Na_2_CO_3_. After 48 h of incubation at 37 °C, the plates were stained using a 0.3% (w/w) congo red solution for 30 min, then destained using 1 M NaCl for 30 min to observe the hydrolytic zones.

### Flocculating activity assay

The flocculating activity assays were performed according to a previous report, with minor modifications [56]. Briefly, the mixture containing 60 mL of 5 g/L kaolin clay suspension, 100 μL of bioflocculant solution, and 1 mL of 1% CaCl_2_ solution was added into a 100-mL beaker, and then rapidly stirred for 2 min, followed by slow mixing for 1 min. After standing for 1 min, the turbidity (OD_550_) of the supernatant was determined using an ultraviolet spectrophotometer (Unic-7200, Shanghai, China). A control experiment using the same procedure but was instead mixed with the same volume of distilled water was performed. The flocculating activity was calculated by measuring the decrease in turbidity of the supernatant according to the equation: ${\text{Flocculating activity}} = [A-B]/A \times 100\%$, where* A* is the absorbance of the control at a wavelength of 550 nm, and* B* is the absorbance of the sample at a wavelength of 550 nm.

### Selection of the optimal lignocellulosic biomass for bioflocculant production

Agricultural wastes were collected from the Xuzhou suburb of China, including rice bran, corn stover, corn cob, wheat straw, and peanut hull. After crushing into powder, these lignocellulosic biomasses were added into fermentation media, and the flocculating activities were compared to select the suitable agricultural waste for the production of bioflocculant. The composition of the fermentation medium was as follows: 20 g/L of dry power of different agricultural wastes, 3 g/L of yeast extract, 1.3 g/L of K_2_HPO_4_, 0.2 g/L of MgSO_4_·7H_2_O, and supplemented with 10 g/L Na_2_CO_3_. The medium without *B. agaradhaerens* C9 inoculation was used as control to rule out that RBBF-C9 was mainly released from the natural lignocellulosic biomasses. In addition, the medium added with 3 g/L of yeast extract but without biomass was used as blank to rule out that the bioflocculant was mainly converted from yeast extract.

### Effects of rice bran dosages and Na_2_CO_3_ concentrations on RBBF-C9 production

To optimize the fermentation conditions of converting rice bran into RBBF-C9, the effects of various rice bran dosages and Na_2_CO_3_ concentrations on RBBF-C9 production were analyzed. Rice bran at concentrations of 0, 4, 8, 12, 16, 20, 25, and 30 g/L was added into the fermentation medium with 3 g/L of yeast extract as nitrogen source. The Na_2_CO_3_ concentration was adjusted to 0, 1, 2, 4, 6, 8, 10, 12, 14, 16, 18, 20, 25, and 30 g/L in the fermentation medium with 20 g/L of rice bran and 3 g/L of yeast extract. The flocculating activities and yields achieved at different fermentation conditions were compared.

### Enzyme activity assay

The substrates were dissolved in 0.2 M phosphate buffer (pH 5.6–8.9) and 0.2 M NaHCO_3_–Na_2_CO_3_ buffer (pH 8.5–11.3). The fermentation broth was collected after 24 h culture and centrifuged at 10,000 rpm for 10 min. The enzyme activities in the supernatant under different pH levels were assessed to determine the optimal pH range for the activities of xylanase and cellulase.

To determine the time profiles of xylanase and cellulase, the supernatants of fermentation broth obtained at different time points were used as enzyme solution. The cellulase activity was determined according to a previous study with slight modifications [60]. Carboxymethyl cellulose (1%, w/v) was dissolved in 0.2 M NaHCO_3_–Na_2_CO_3_ buffer (pH 10) using as substrate. A reaction mixture containing 150 μL of carboxymethyl cellulose solution and 50 μL of the enzyme solution was incubated at 50 °C for 30 min, followed by the addition of 100 μL of 1 M NaOH and 150 μL dinitrosalicylic acid (DNS) to stop the reaction. After boiling for 5 min, 550 μL of H_2_O was added to the mixture, and the OD_540_ was measured using a Unic-7200 spectrophotometer. The enzyme solution inactivated by boiling was used as control. One unit of cellulase activity was defined as the amount of enzyme liberating 1 μM of glucose per minute.

The amount of reducing sugars produced from 1% (w/v) oat spelts xylan (sigma) dissolved in 0.2 M NaHCO_3_–Na_2_CO_3_ buffer (pH 10.0) was determined in the xylanase activity assay. 50 μL of the enzyme solution and 150 μL of the oat spelts xylan solution were mixed and incubated at 50 °C for 30 min. The DNS method was performed to determine the amount of reducing sugars that was released, using xylose as a standard sample. The inactivated enzyme solution was used as control. One unit of xylanase was defined as the enzyme amount that released 1 μM of xylose per minute.

### Production and extraction of the bioflocculant product

*Bacillus agaradhaerens* C9 cells were cultured in 25 mL of the seed medium at 37 °C for 12 h. 1 mL of seed culture was inoculated into 500-mL Erlenmeyer flasks containing 100 mL of optimized fermentation medium. The optimal fermentation medium contained 20 g/L of untreated dry rice bran, 3 g/L of yeast extract, 1.3 g/L of K_2_HPO_4_, 0.2 g/L of MgSO_4_·7H_2_O, and supplemented with 20 g/L of Na_2_CO_3_. After 24 h of incubation with 180-rpm shaking at 37 °C, the cells and rice bran residues in fermentation broth were removed by centrifugation at 12,000 rpm at 4 °C for 10 min. The bioflocculant RBBF-C9 was precipitated from the supernatant by adding two volumes of cold absolute ethanol. The resulting RBBF-C9 product was centrifuged at 10,000 rpm for 5 min, washed with 75% ethanol twice, and lyophilized to dryness using a BETA 1-8 LD plus Freeze Dryer (Christ, Germany).

### Characteristics of bioflocculant RBBF-C9

The general properties of RBBF-C9 were determined according to a previous study [16]. The content of polysaccharides in RBBF-C9 was analyzed by using the phenol–sulfuric acid method using glucose as the standard [68]. The Bradford method was performed to determine the protein content with bovine serum albumin as the standard [69]. Gel permeation chromatography was carried out to identify the molecular weight of RBBF-C9 using a Hitachi L-6200 system controller [42]. Elemental analysis was performed using an elemental analyzer (Elementar vario EL, Germany). The functional groups of RBBF-C9 were analyzed by using a fourier transform infrared (FTIR) spectroscopy (Bruker Tensor 27, Germany) in a wavelength range from 600 to 4000 cm^−1^.

### Culture and harvest of *C. minutissima* UTEX2341 cells

The oil algae *C. minutissima* UTEX2341 was stored in our laboratory and cultured in IM medium as described in a previous study [51, 61]. *C. minutissima* UTEX2341 was cultured in 500-mL Erlenmeyer flasks containing 200 mL of liquid IM medium at 24 °C, with a 14-/10-h light/dark cycle for 2 weeks.

The extracted RBBF-C9 was resolved in deionized water to prepare a 5 g/L RBBF-C9 solution, and the pH of this RBBF-C9 solution was adjusted to 7.0 using 1 M NaOH or HCl solution. To evaluate the flocculating efficiency of RBBF-C9 to *C. minutissima* UTEX2341 cells, 60 mL of the algal culture broth was poured into 100-mL beakers, added with RBBF-C9 solution to different concentrations, and mixed with 0.8 mL 10% CaCl_2_. The mixtures were rapidly stirred for 5 min, followed by slow mixing for 1 min. After standing for 10 min, the supernatant was collected to determine the OD_675_ by using a Unic-7200 spectrophotometer (Shanghai, China). Algal culture broth supplied with same volume of deionized water and subjected to the same procedure was used as control. The flocculating efficiency of the *C. minutissima* UTEX2341 cells was calculated according to the following equation: ${\text{Flocculating efficiency}} = (X-Y)/X \times 100\%$, where* X* is the OD_675_ value of the control, and* Y* is the OD_675_ value of the supernatant of the flocculated sample.

## Additional file

**Additional file 1: Figure S1.** Fourier transform infrared spectroscopy of bioflocculant RBBF-C9.

## Abbreviations

RBBF-C9: the bioflocculant converted from untreated rice bran by *Bacillus agaradhaerens* C9
CMC: carboxymethyl cellulose
DNS: 3, 5-dinitrosalicylic acid
CGMCC: China General Microbiological Culture Collection Center
GPC: gel permeation chromatography
FTIR: fourier transform infrared spectroscopy
