# Supplementary figures and images for: Valorization of untreated rice bran towards bioflocculant using a lignocellulose-degrading strain and its use in microalgal biomass harvest
Source: Biotechnol Biofuels. 2017 Apr 13;10:90. doi: 10.1186/s13068-017-0780-6 (PMC5390349; doi:10.1186/s13068-017-0780-6)

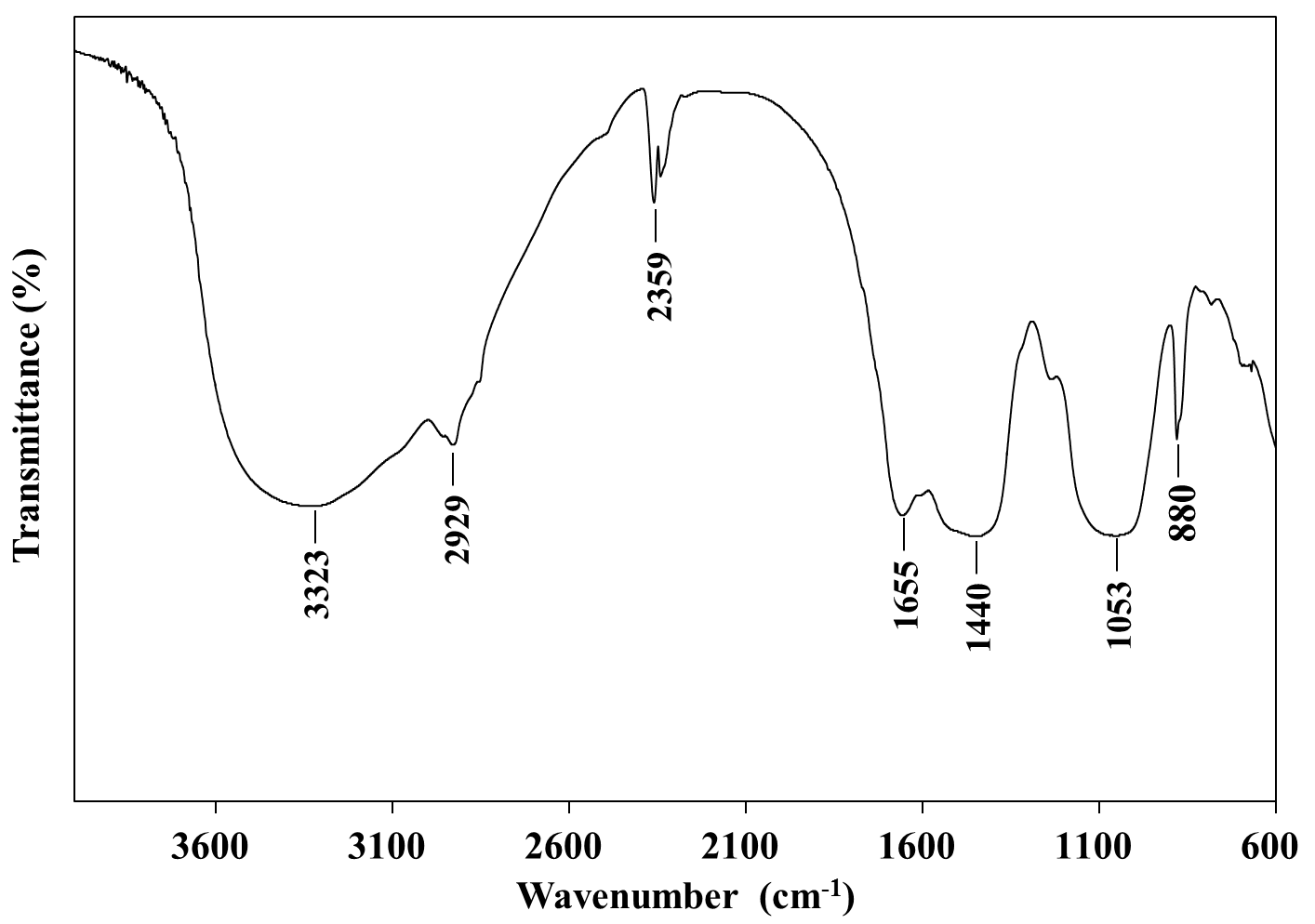


Figure - S1

Supplement: Supplementary file 1 — Additional file 1: Figure S1. Fourier transform infrared spectroscopy of bioflocculant RBBF-C9. [file 13068_2017_780_MOESM1_ESM.docx]
